# Supplementary material for: Dr. Google: Physicians—The Web—Patients Triangle: Digital Skills and Attitudes towards e-Health Solutions among Physicians in South Eastern Poland—A Cross-Sectional Study in a Pre-COVID-19 Era
Source: Int J Environ Res Public Health. 2023 Jan 5;20(2):978. doi: 10.3390/ijerph20020978 (PMC9858975; doi:10.3390/ijerph20020978)
Supplement: Supplementary file 1 [file ijerph-20-00978-s001.zip › Supplementary S1_Questionnaire.pdf]

E-health* in the opinion of physicians

Survey questionnaire

We invite you to participate in the research project entitled **THE USE OF E-HEALTH SOLUTIONS AND DIGITAL LITERACY OF PHYSICIANS, NURSES AND PATIENTS**,, conducted at the Institute of Nursing and Health Sciences at the Medical Faculty of the University of Rzeszów.

Our goal is to identify readiness to use modern e-health tools in the context of the digital literacy of physicians, nurses and patients. We are particularly interested in your personal experience and opinion on this subject.

The results of the study are anonymous and will only be used for scientific purposes in the collective study. We kindly ask you to fill in the questionnaire below, which is tantamount to agreeing to take part in the study.

*E-Health is the use of information and communication technologies in health-related tools, devices, services and solutions.

1. **How often do you use the following devices and solutions:**

**A. In private life**						**B. In professional work**					
		Often	Sometimes	Never	N/A			Often	Sometimes	Never	N/A
A.1.1	computer					B.1.1.	computer				
A.1.2	tablet					B.1.2.	tablet				
A.1.3	smartphone					B.1.3.	smartphone				
A.1.4	email					B.1.4	email				
A.1.5	mobile apps					B.1.5	mobile apps				

2. **How often do you use the internet?**

€ several times a day
€ every day
€ several times a week
€ once a week
€ I do not use

Section: DIGITAL LITERACY

3. **Please assess your ability to use devices/solutions from pyt. 1 and the Internet:**

€ very good
€ good
€ sufficient
€ weak
€ very weak

4. **Please answer the following:**

		Totally agree	I agree	I have no opinion	I disagree	I completely disagree
4.1	I feel prepared to handle e-health solutions related to my professional work					
4.2.	I would benefit from additional trainings /courses in the field of shaping digital competences					
4.3.	Today's physician training keeps pace with the digital challenges of the twenty-first century.					
4.4.	There should be more subjects in medical studies that shape digital competences					

Section: THE INFLUENCE OF THE INTERNET / NEW TECHNOLOGIES ON HEALTHCARE AND MODERN LIFE

5. **Do you think the Internet will revolutionize healthcare?**

€ Rather yes
€ Yes
€ I do not know
€ No
€ Rather not

6. **What is your feeling about new technologies in the life of modern man?**

€ Fascinate me
€ I'm interested in it
€ They are helpful
€ I have no opinion
€ I am concerned about this phenomenon
€ I am terrified of this phenomenon

Section: DR GOOGLE

7. **Based on your own observations, please estimate the percentage of your patients accessing online health materials in a month:**

€ <1%
€ 1-2%
€ 3-5%
€ 6-10%
€ >10%
€ it is difficult for me to estimate

8. **What do you think about the overall quality of the health information you can find on the Internet?**

€ reliable

*more than 1 answer can be indicated

- € usually reliable
- € sometimes unbelievable
- € unbelievable
- € I have no opinion

9. **Have any of your patients experienced health benefits as a result of accessing online materials?**
   - € often
   - € sometimes
   - € seldom
   - € never
   - € I do not have such knowledge

10. **How do you assess the effects of patients using health knowledge from the Internet?**
    - € very positive
    - € positively
    - € meaningless
    - € negative
    - € very negativee

Section: RECOMMENDATION OF E-HEALTH SOLUTIONS

11. **To what extent would you recommend electronic solutions to your patients to support treatment and self-control in the following areas:**

		I already recommend to my patients	I would recommend it to my patients	I do not use
11.1.	remote monitoring of basic vital signs (pressure, heart rate, temperature, glucose level)			
11.2.	obtaining the results of laboratory tests via the Internet			
11.3.	arranging medical appointments via the Internet			
11.4.	using a mobile application that facilitates the analysis of research results			
11.5.	using a mobile application that is a knowledge base on health-related topics			
11.6.	using a mobile application, which is a mobile database of medicines			
11.7.	using a mobile application that reminds you to take medication			
11.8.	using video consultations with a doctor / nurse / midwife as a support for the treatment process			
11.9.	another (own response)			

Section: EVALUATION OF E-HEALTH SOLUTIONS

*more than 1 answer can be indicated

12. **Please specify how you assess the following e-health solutions:**

		Very important	Relevant	Not important	Completely irrelevant	I have no opinion
12.1.	Easy and quick access to the patient's medical records in electronic form					
12.2.	Possibility of writing electronic prescriptions					
12.3.	Possibility of writing electronic sick leaves					
12.4.	Possibility to write out electronic referrals					
12.5.	Using the electronic drug database					
12.6.	Ability to remotely refer patients to other specialists or hospitals					
12.7.	Solutions to facilitate the sending/exchange of clinical results					
12.8.	Solutions for remote care					
12.9.	Increasing the share of digital solutions supporting the treatment and self-control of the patient's health					
12.10.	Possibility of maintaining comprehensive control over the facility and tracking generated costs, personnel management (graphics, schedules)					
12.11.	Possibility to conduct scientific research					
12.12.	Other (own response)					

SOCJODEMOGRAPHIC section

13. **Sex**

€ Woman
€ Man

14. **Year of birth: ....**

15. **Specialization ....**

16. **Workplace:**

€ hospital
€ primary care clinic
€ private practice
€ family practice
€ ambulance
€ home hospice
€ hospice visits

*more than 1 answer can be indicated

€ 24/7 medical assistance

€ diagnostic laboratory

€ other, what? ....

17. **On average, how many patients do you see in a month?**

€ <50

€ 50-100

€ 100-200

€ >200

Thank you for completing the survey!

*more than 1 answer can be indicated
