# Supplementary material for: Dr. Google: Physicians—The Web—Patients Triangle: Digital Skills and Attitudes towards e-Health Solutions among Physicians in South Eastern Poland—A Cross-Sectional Study in a Pre-COVID-19 Era
Source: Int J Environ Res Public Health. 2023 Jan 5;20(2):978. doi: 10.3390/ijerph20020978 (PMC9858975; doi:10.3390/ijerph20020978)
Supplement: Supplementary file 1 [file ijerph-20-00978-s001.zip › Supplementary S2_Freqencies.pdf]

Supplementary S2

Frequencies

Table S1. The proportion of respondents by gender

Gender	N	%	% Cumulative
female	168	54.7	54.7
male	139	45.3	100.0
Total	307	100.0	

Table S2. The proportion of respondents by age

Age	N	%	% Cumulative
26	2	0.7	0.7
27	8	2.6	3.3
28	13	4.2	7.5
29	24	7.8	15.3
30	14	4.6	19.9
31	22	7.2	27.0
32	11	3.6	30.6
33	15	4.9	35.5
34	7	2.3	37.8
35	3	1.0	38.8
36	5	1.6	40.4
37	5	1.6	42.0
38	5	1.6	43.6
39	9	2.9	46.6
40	3	1.0	47.6
41	4	1.3	48.9
42	10	3.3	52.1
43	4	1.3	53.4
44	5	1.6	55.0
45	3	1.0	56.0
46	2	0.7	56.7
47	11	3.6	60.3
48	3	1.0	61.2
49	9	2.9	64.2
50	6	2.0	66.1
51	11	3.6	69.7
52	12	3.9	73.6
53	4	1.3	74.9
54	2	0.7	75.6
55	3	1.0	76.5
57	3	1.0	77.5
58	3	1.0	78.5
59	11	3.6	82.1
60	6	2.0	84.0
61	3	1.0	85.0
62	2	0.7	85.7

63	4	1.3	87.0
64	3	1.0	87.9
65	7	2.3	90.2
66	3	1.0	91.2
67	4	1.3	92.5
68	1	0.3	92.8
71	6	2.0	94.8
72	5	1.6	96.4
73	2	0.7	97.1
74	4	1.3	98.4
75	1	0.3	98.7
77	3	1.0	99.7
78	1	0.3	100.0
Total	307	100.0	

Table S3. The proportion of respondents by specialization

Specialization	N	%	% Cumulative
Anaesthesiology	7	2.3%	2.3%
Vascular surgery	1	0.3%	2.6%
General surgery	3	1.0%	3.6%
Internal	27	8.8%	12.4%
Dermatologist	5	1.6%	14.0%
Genecology	11	3.6%	17.6%
Cardiology	7	2.3%	19.9%
Family medicine	136	44.3%	64.2%
Neurology	18	5.9%	70.0%
Ophthalmologist	5	1.6%	71.7%
Orthopaedist	28	9.1%	80.8%
Paediatrician	32	10.4%	91.2%
Pulmonology	6	2.0%	93.2%
Rheumatology	2	0.7%	93.8%
Resident	6	2.0%	95.8%
Dentistry	3	1.0%	96.7%
In the course of Spec. - Genecology	3	1.0%	97.7%
In the course of Spec. - Med. Genus.	3	1.0%	98.7%
In the course of Spec. - Oncology	3	1.0%	99.7%
W + BI1391	1	0.3%	100.0%
Total	307	100.0%	

Table S4. Workplace of surveyed physicians

Workplace	I do not confirm		I confirm	
	N	%	N	%
hospital	200	65.1%	107	34.9%
primary healthcare	57	18.6%	250	81.4%
private practice	253	82.4%	54	17.6%
family practice	303	98.7%	4	1.3%
ambulance	305	99.3%	2	0.7%
home hospice	307	100.0%	0	0.0%
hospice visits	307	100.0%	0	0.0%
24/7 medical assistance	289	94.1%	18	5.9%
diagnostic laboratory	298	97.1%	9	2.9%

Table S5. No. of patients seen on average per month.

	N	%	% Cumulative
< 50	38	12.4	12.4
50-100	86	28.0	40.4
100-200	35	11.4	51.8
> 200	148	48.2	100.0
Total	307	100.0	

Table S6. Frequency of Internet use.

	N	%	% Cumulative
several times a day	205	66.8	66.8
every day	61	19.9	86.6
several times a week	17	5.5	92.2
once a week	17	5.5	97.7
I do not use	7	2.3	100.0
Total	307	100.0	

Physicians' Digital literacy

Table S7. Self-assessment of the ability to use mobile devices / solutions.

	N	%	% Cumulative
very good	103	33.6	33.6
good	100	32.6	66.1
sufficient	75	24.4	90.6
weak	22	7.2	97.7
very weak	7	2.3	100.0
Total	307	100.0	
