# Supplementary material for: Dr. Google: Physicians—The Web—Patients Triangle: Digital Skills and Attitudes towards e-Health Solutions among Physicians in South Eastern Poland—A Cross-Sectional Study in a Pre-COVID-19 Era
Source: Int J Environ Res Public Health. 2023 Jan 5;20(2):978. doi: 10.3390/ijerph20020978 (PMC9858975; doi:10.3390/ijerph20020978)
Supplement: Supplementary file 1 [file ijerph-20-00978-s001.zip › Supplementary S3_Correlations.pdf]

Supplementary S3

Correlations

Table S8. Correlations between using devices in physicians' private and professional life, and digital literacy and e-Health indicators.

		Digital literacy – own skills		Digital literacy – the need for training		The impact of the Internet / new technologies on healthcare and modern life		Recommendatio n of e-Health solutions		Evaluation of e-Health solutions - the patient		Evaluation of e-Health solutions - medical facility	
		**Mean**	**SD**	**Mean**	**SD**	**Mean**	**SD**	**Mean**	**SD**	**Mean**	**SD**	**Mean**	**SD**
						In private life							
computer	often	-0.20	0.90	0.21	0.92	-0.14	1.02	−0.20	0.99	−0.24	0.78	−0.01	0.98
	sometimes	0.37	0.95	−0.40	0.99	0.18	0.80	0.35	0.92	0.32	1.01	0.05	1.01
	never	0.18	1.46	−0.52	1.49	0.52	1.27	0.42	0.81	1.80	2.04	−0.36	1.27
	N/A	1.36	2.90	−0.37	0.79	2.18	1.45	1.22	0.45	1.14	1.94	0.31	1.68
tablet	often	−0.42	0.82	−0.09	0.73	−0.37	0.73	−0.40	0.93	−0.55	0.57	−0.05	0.84
	sometimes	−0.03	0.92	0.16	1.03	−0.15	0.89	−0.09	0.92	−0.11	0.77	−0.04	0.94
	never	0.27	0.88	−0.05	1.15	0.31	1.03	0.34	0.95	0.42	1.17	0.17	1.17
	N/A	0.16	1.66	−0.20	0.77	0.32	1.42	0.03	1.25	0.20	1.19	−0.40	0.72
smartphone	often	−0.07	0.91	0.20	0.86	−0.13	0.93	−0.11	0.97	−0.19	0.79	0.01	0.90
	sometimes	0.46	1.16	−0.87	1.30	0.69	0.83	0.43	0.89	0.89	1.39	−0.26	1.28
	never	0.19	1.21	−1.26	0.80	0.49	1.16	0.60	1.18	0.97	1.36	−0.04	1.39
	N/A	0.90	2.54	−0.07	0.45	1.39	1.73	0.71	0.69	1.26	1.43	0.95	2.00
e–mail	often	−0.16	0.92	0.18	0.85	−0.17	0.94	−0.18	0.95	−0.25	0.71	−0.07	0.83
	sometimes	0.36	0.82	−0.24	1.23	0.20	1.02	0.23	0.97	0.07	0.92	0.30	1.15
	never	0.36	1.31	−1.18	0.65	0.78	0.83	0.76	0.94	1.72	1.40	−0.48	1.20
	N/A	0.46	1.85	−0.12	1.23	0.93	1.07	1.05	0.58	1.34	1.31	0.59	1.95
mobile apps	often	−0.24	0.94	0.30	0.87	−0.40	0.77	−0.25	1.04	−0.37	0.70	0.08	0.87
	sometimes	0.24	0.79	−0.11	1.04	0.46	1.10	0.16	0.66	0.19	0.81	−0.10	1.08
	never	0.42	1.13	−0.88	0.86	0.55	0.85	0.60	1.01	0.93	1.40	−0.26	1.16
	N/A	0.38	1.68	−0.50	0.75	0.77	1.23	0.34	1.14	0.79	1.24	0.56	1.42
						At work							
computer	often	−0.12	0.89	0.10	0.98	−0.15	0.93	−0.09	0.97	−0.18	0.79	0.02	0.95
	sometimes	0.80	1.19	−0.69	0.99	0.93	0.95	0.62	0.99	1.12	1.18	−0.28	1.09
	never	0.14	1.00	−0.44	0.70	0.44	0.63	0.16	1.10	0.60	1.67	0.36	1.43
	N/A	4.70	–	0.51	–	3.85	–	1.73	–	3.39	–	−1.62	–
tablet	often	−0.66	1.08	−0.58	0.77	−0.36	0.45	−0.81	1.09	−0.64	0.74	−0.04	0.66
	sometimes	−0.97	0.74	0.06	0.71	−0.47	0.68	0.12	0.45	0.05	0.84	0.51	1.33
	never	0.12	0.87	0.01	1.06	0.06	0.96	0.03	0.98	0.05	1.02	−0.02	1.00
	N/A	0.07	1.31	0.12	0.79	0.04	1.34	0.09	1.15	−0.06	1.01	−0.14	0.85
smartphone	often	−0.22	1.00	0.24	1.05	−0.32	0.86	−0.26	1.06	−0.31	0.80	0.02	0.84
	sometimes	0.06	0.62	0.06	0.81	0.19	1.00	0.09	0.50	−0.06	0.62	0.03	1.08
	never	0.27	1.07	−0.57	0.97	0.32	0.91	0.21	1.15	0.65	1.34	0.01	1.28
	N/A	0.48	1.45	−0.13	0.65	0.59	1.43	0.83	0.78	0.34	1.14	−0.26	0.75
e–mail	often	−0.40	0.92	0.37	0.88	−0.14	1.07	−0.38	1.03	−0.42	0.66	−0.06	0.79
	sometimes	−0.03	0.83	0.04	0.93	−0.17	0.73	0.04	0.78	−0.17	0.70	−0.08	0.92
	never	0.25	0.96	−0.49	1.04	0.24	1.04	0.18	1.07	0.50	1.24	0.21	1.26
	N/A	1.45	1.30	−0.02	0.99	1.00	1.43	1.13	0.84	1.38	1.12	−0.19	1.12
mobile apps	often	−0.34	0.96	0.37	1.11	−0.32	0.93	−0.44	1.03	−0.47	0.68	−0.13	0.94
	sometimes	0.03	0.97	0.27	0.92	−0.08	0.93	0.10	0.84	−0.01	0.75	0.08	0.84
	never	0.09	0.89	−0.47	0.85	0.19	0.91	0.09	1.02	0.25	1.24	−0.03	1.18

N/A	0.56	1.31	−0.30	0.69	0.54	1.44	0.55	0.97	0.43	1.14	0.15	1.00

The higher the mean value, the lower the digital literacy, the lower the evaluation of the Internet impact, the lower the recommendation, and the lower the evaluation of e-health solutions

Table S9. Corelations between physicians' age, digital literacy and e-Health indicators.

**Indicator**	rho	*p*	N
Digital literacy – own skills	0.189	0.0009	307
Digital literacy – the need for training	−0.517	0.0000	307
The influence of the Internet / new technologies on healthcare and	0.291	0.0000	307
Recommendation of e-Health solutions	0.269	0.0000	307
Evaluation of e-health solutions - the patient	0.396	0.0000	307
Evaluation of e-health solutions - medical facility	−0.150	0.0087	307

Physicians' opinion about online health information (Dr Google)

Table S10. % of patients accessing online health information within a month.

	**N**	**%**	**% Cumulative**
< 1%	12	3.9	3.9
1–2%	19	6.2	10.1
3–5%	52	16.9	27.0
6–10%	43	14.0	41.0
> 10%	102	33.2	74.3
difficult to calculate	79	25.7	100.0
Total	307	100.0	

Table S11. Physicians' opinion on the overall quality of online health information.

	**N**	**%**	**% Cumulative**
reliable	10	3.3	3.3
usually reliable	55	17.9	21.2
sometimes reliable	164	53.4	74.6
unreliable	57	18.6	93.2
no opinion	21	6.8	100.0
Total	307	100.0	

Table S12. Physicians' opinion on patients' health benefits related to online health knowledge.

	**N**	**%**	**% Cumulative**
often	16	5.2	5.2
sometimes	106	34.5	39.7
seldom	73	23.8	63.5
never	13	4.2	67.8
I do not have such knowledge	99	32.2	100.0
Total	307	100.0	

Table S13. Physicians' assessment of patients' use of online health knowledge.

	N	%	% Cumulative
very positive	9	2.9	2.9
positive	55	17.9	20.8
meaningless	68	22.1	43.0
negative	128	41.7	84.7
very negative	47	15.3	100.0
Total	307	100.0	

Table S14. Physicians' opinion that the Internet will revolutionize healthcare.

	N	%	% Cumulative
rather yes	116	37.8	37.8
yes	123	40.1	77.9
I do not know	52	16.9	94.8
no	4	1.3	96.1
rather not	12	3.9	100.0
Total	307	100.0	

Table S15. Physicians' feelings about new technologies in the modern life.

	N	%	% Cumulative
Fascinates me	25	8.1	8.1
I'm interested in this	72	23.5	31.6
It's helpful	176	57.3	88.9
I have no opinion	6	2.0	90.9
I am concerned about this phenomenon	19	6.2	97.1
I am terrified of this phenomenon	9	2.9	100.0
Total	307	100.0	

Table S16. Opinion about the general quality of online health information.

	N	%	% Cumulative
reliable	10	3.3	3.3
usually reliable	55	17.9	21.2
sometimes reliable	164	53.4	74.6
un reliable	57	18.6	93.2
no opinion	21	6.8	100.0
Total	307	100.0	

Table S17. Opinion on patients' experience of health benefits as a result of access to online health information.

	N	%	% Cumulative
often	16	5.2	5.2
sometimes	106	34.5	39.7
seldom	73	23.8	63.5
never	13	4.2	67.8
I do not have such a knowledge	99	32.2	100.0
Total	307	100.0	

Table S18. An assessment of patients' use of online health knowledge

	N	%	% Cumulative
very positive	9	2.9	2.9
positive	55	17.9	20.8
meaningless	68	22.1	43.0
negative	128	41.7	84.7
very negative	47	15.3	100.0
Total	307	100.0	

Dr. Google and indicators

The conducted analyses also showed that respondents working in the diagnostic laboratory had higher digital skills (−0.23) than those not working there (0.01). Physicians employed in the hospital presented a higher need for training (0.16) but hospital personnel (−0.26), not working in a primary healthcare (−0.23), conducting a private practice (−0.44), providing 24/7 medical assistance (−0.54), and working in the diagnostic laboratory (−0.89), rated higher the impact of the Internet and new technologies on healthcare and the modern life. The recommendation of e-Health solutions was also higher among respondents employed in hospitals (−0.30) and physicians not working in primary healthcare (−0.36). The assessment of e-Health solutions in relation to the patient was higher in hospital personnel (−0.32), not working in a primary healthcare (−0.33), and working in a diagnostic laboratory (−0.35). The evaluation of e–health solutions in relation to a medical facility was higher in respondents not working in emergency services (−0.02) and people working in 24–hour medical assistance (−0.37).

Table S19. Correlations between type of workplace, and digital literacy and other e-Health indicators (means)

Workplace		Digital competence - skills		Digital competences - the need for training		Assessment of the impact of the Internet and new technologies on healthcare and the life of modern man		Recommendation of e-health solutions		Evaluation of e-health solutions - patient		Evaluation of e-health solutions - medical facility	
		Mean	SD	Mean	SD	Mean	SD	Mean	SD	Mean	SD	Mean	SD
hospital	no	0.04	1.09	−0.09	1.00	0.14	1.05	0.16	1.01	0.17	1.13	0.06	1.08
	yes	−0.08	0.80	0.16	0.99	−0.26	0.84	−0.30	0.91	−0.32	0.59	−0.10	0.83
primary healthcare	no	−0.03	0.86	0.18	1.26	−0.23	0.96	−0.36	0.90	−0.33	0.60	−0.13	0.87
	yes	0.01	1.03	−0.04	0.93	0.05	1.00	0.08	1.01	0.07	1.06	0.03	1.03
private practice	no	0.01	1.04	−0.03	0.96	0.09	1.05	0.04	1.01	0.05	1.06	0.00	1.04
	yes	−0.06	0.81	0.14	1.16	−0.44	0.56	−0.19	0.91	−0.23	0.60	0.00	0.82

family practice	no	0.00	1.00	0.01	0.99	−0.01	1.00	0.00	1.01	0.01	1.00	0.00	1.00
	yes	−0.10	0.87	−0.72	1.31	0.59	1.04	0.13	0.40	−0.62	0.64	0.25	1.02
ambulance	no	−0.01	1.00	0.00	1.00	0.01	1.00	0.01	1.00	0.00	1.00	−0.02	0.99
	yes	0.89	0.00	−0.64	0.00	−1.10	0.00	−1.33	0.00	−0.36	0.00	2.34	0.00
24/7 medical assistance	no	0.03	1.01	0.00	1.01	0.03	1.00	0.01	1.02	0.00	0.98	0.02	1.03
	yes	−0.44	0.80	0.03	0.79	−0.54	0.76	−0.11	0.63	0.01	1.34	−0.37	0.21
diagnostic laboratory	no	0.01	1.01	−0.01	1.01	0.03	1.00	0.00	1.01	0.01	1.01	0.01	1.01
	yes	−0.23	0.21	0.40	0.57	−0.89	0.31	0.13	0.37	−0.35	0.35	−0.19	0.72

Table S20. Correlations between type of workplace, and digital literacy and other e-Health indicators (t-test)

**Workplace**		**Digital competence - skills**	**Digital competences - the need for training**	**Assessment of the impact of the Internet and new technologies on healthcare and the life of modern man**	**Recommendation of e-health solutions**	**Evaluation of e-health solutions - patient**	**Evaluation of e-health solutions - medical facility**
hospital	t	1.129	−2.080	3.663	3.970	5.100	1.443
	p	0.2599	0.0383	0.0003	0.0001	0.0000	0.1501
primary healthcare	t	−0.230	1.231	−1.970	−3.020	−3.863	−1.175
	p	0.8183	0.2223	0.0498	0.0027	0.0002	0.2430
private practice	t	0.545	−1.105	5.279	1.532	1.900	−0.033
	p	0.5869	0.2699	0.0000	0.1265	0.0584	0.9741
family practice	t	0.211	1.442	−1.191	−0.257	1.252	−0.506
	p	0.8334	0.1503	0.2345	0.7976	0.2114	0.6133
ambulance	t	−1.258	0.914	1.557	1.891	0.509	−3.369
	p	0.2095	0.3612	0.1204	0.0595	0.6110	0.0009
24/7 medical assistance	t	1.911	−0.110	2.387	0.751	−0.036	5.014
	p	0.0569	0.9126	0.0176	0.4605	0.9716	0.0000
diagnostic laboratory	t	2.600	−1.224	7.818	−1.010	2.730	0.566
	p	0.0160	0.2219	0.0000	0.3324	0.0176	0.5717

t−Student’s t−test
